# Moving a missing hand: children born with below elbow deficiency can enact hand grasp patterns with their residual muscles

**DOI:** 10.1186/s12984-024-01306-z

**Published:** 2024-01-23

**Authors:** Justin J. Fitzgerald, Marcus A. Battraw, Michelle A. James, Anita M. Bagley, Jonathon S. Schofield, Wilsaan M. Joiner

**Affiliations:** 1 Department of Biomedical Engineering, University of California, Davis, CA USA; 2 Department of Neurobiology, Physiology and Behavior, University of California, 1 Shields Avenue, Davis, CA 95616 USA; 3 Clinical and Translational Science Center, University of California Davis Health, Sacramento, CA USA; 4 Department of Mechanical and Aerospace Engineering, University of California, Davis, CA USA; 5 Shriners Children’s Northern California, Sacramento, CA USA; 6 Department of Orthopaedic Surgery, University of California Davis Health, Sacramento, CA USA; 7 Department of Neurology, University of California Davis Health, Sacramento, CA USA

## Abstract

Children with a unilateral congenital below elbow deficiency (UCBED) have one typical upper limb and one that lacks a hand, ending below the elbow at the proximal/mid forearm. UCBED is an isolated condition, and affected children otherwise develop normal sensorimotor control. Unlike adults with upper limb absence, the majority of whom have an acquired loss, children with UCBED never developed a hand, so their residual muscles have never actuated an intact limb. Their ability to purposefully modulate affected muscle activity is often assumed to be limited, and this assumption has influenced prosthetic design and prescription practices for this population as many modern devices derive control signals from affected muscle activity. To better understand the motor capabilities of the affected muscles, we used ultrasound imaging to study 6 children with UCBED. We examined the extent to which subjects activate their affected muscles when performing mirrored movements with their typical and missing hands. We demonstrate that all subjects could intentionally and consistently enact at least five distinct muscle patterns when attempting different missing hand movements (e.g., power grasp) and found similar performance across affected and typically developed limbs. These results suggest that although participants had never actuated the missing hand they could distinctively and consistently activate the residual muscle patterns associated with actions on the unaffected side. These findings indicate that motor control still develops in the absence of the normal effector, and can serve as a guide for developing prostheses that leverage the full extent of these children’s motor control capabilities.

**Supplementary Information:**

The online version contains supplementary material available at 10.1186/s12984-024-01306-z.

## Introduction

It is commonly believed that children born without a hand (unilateral congenital below elbow deficiency, UCBED) have diminished or absent cerebral sensorimotor representations of their missing limb. Imaging of the cerebral cortex has provided evidence that the missing hand’s territory is utilized by adjacent regions representing the residual arm, and this phenomenon is dependent on the use of the residual arm in compensating during activities of daily living [1, 2]. Furthermore, it has been shown that cortical representations of missing digits may be diminished in this population and cortical activation patterns bear little resemblance to those observed in individuals with acquired hand loss and able-bodied control cohorts [3]. Although genetics likely support the preliminary formation of a hand representation [4], it is suggested that early-life experiences and the accompanying sensorimotor input from the hand are critical to the maturation of its typical and functional organization [3].

In many ways, the prosthetic options and the standard of clinical care for children with UCBED have been shaped by the assumption that a child’s diminished representation of the missing hand is detrimental to their perceptions of prostheses, as well as their willingness and ability to use them. Children with UCBED learn compensatory strategies to perform activities of daily living from an early age and it is often observed that they do not feel a sense of loss, although they may feel different from their peers [5, 6]. Even though cosmetic factors associated with wearing a prosthesis may help with social integration, functionally, for children with UCBED these devices seldom provide an improved quality of life [7]. In contrast, older children who lose a limb later in life will more readily use a prosthesis, presumably because they have experienced the loss of function and have a more mature sensorimotor representation of their now missing limb [8]. Considering these assumptions, prevailing opinion suggests that if a child with UCBED learns to handle a prosthesis at a young age, the prosthesis might be better incorporated into the body scheme [9] and thus the age at which a prosthesis is first used is viewed as a critical factor in life-long prosthesis acceptance [10, 11]. Consequently, device prescription can occur as young as 2 months of age [9] with the intention of promoting the development of motor programs in the sensorimotor cortices that include prosthesis use [12, 13].

However, regardless of current best practices shaping the age of first prescription, an estimated 35–45% of pediatric prostheses will be abandoned [14] as many conventional devices offer limited functional benefit and may even hinder during the performance of daily activities [7]. Encouragingly, in recent years there has been an acceleration in prosthetic mechatronic technologies resulting in child sized devices that more closely resemble the form and function of intact hands [15]. However, control of these hands relies on the ability of children to skillfully contract their affected muscles which is then mapped to the device opening, closing, and/or toggling between grasping movements, using surface electromyography (sEMG). For adults, advanced commercially available sEMG systems use pattern recognition algorithms to decode affected muscle electrical-activity and can link the users intended missing limb movements to prosthetic movements [16–19]. Both in the laboratory and in real-world prostheses, this control technique has largely been shown to improve adult-user function and control over mechatronic prostheses [20–22].

Similar control systems have yet to be translated for children with UCBED. This is due, at least in part, to the common assumption that since these children have never had an intact limb on the affected side and have diminished cortical representation, this limits the function and movement repertoires of their affected muscles [23]. Thus, the perception is that the user cannot generate the requisite diversity in muscle responses to activate advanced prosthetic control systems that are designed to decode a variety of motor intentions [24, 25]. Yet these assumptions have yet to be rigorously investigated. In small cohorts of adults with UCBED (N = 1–2), commercially available pattern recognition control systems show that 3–4 missing hand movements could be reliably detected and classified for prosthetic control. However, studies of the motor capabilities of affected muscles in larger cohorts of people with UCBED (especially in comparison to the unaffected limb), or cohorts including children, have not been performed.

Despite the evidence of diminished cortical representations in children with UCBED, it is known that some degree of control over the affected muscles remains. For example, to operate conventional mechatronic (myoelectric) prosthesis, these children learn to isolate and contract groups of residual muscles (typically the volar flexor muscle mass and the dorsal extensor muscle mass). Furthermore, although cortical activity may differ in this population, this data does not necessarily imply that neural sensorimotor mechanisms of the missing hand are wholly absent. In fact, more than 20% of children born without limbs experience phantom sensations of their missing appendage, and this phenomenon can remain throughout adulthood [26, 27]. Furthermore, studies pairing fMRI and transcranial magnetic stimulation with perceptual and behavioral data suggest that body parts that have never developed can still be represented in sensory and motor cortical areas [26]. It has been proposed that both genetic and epigenetic factors, such as the habitual observation of other people moving their limbs, may contribute to the conscious experience of moving a phantom limb [26]. However, the connections between the perceptions of moving a physically absent hand (motor imagery) and the resulting muscle responses in the residual limb remain poorly understood.

Here, we employed an emerging technique known as sonomyography [26, 27], to examine the motor capabilities of the affected muscle in children with UCBED. Sonomyography combines ultrasound imaging and machine learning, to interpret motor intent of the hand and wrist from spatiotemporal motion patterns of forearm muscles. We used this approach to investigate the extent to which affected forearm musculature responds and represents distinct missing hand motions when children with UCBED attempt to move their missing limbs. In addition, due to the unique characteristics of this participant population, we also compared performance across the affected and typically developed limbs.

## Materials and methods

### Study design

We recruited six pediatric participants (age range 6–20) at Shriners Hospitals for Children—Northern California (Sacramento, CA, USA). Subject-specific details are available in Tables 1 and 2. All participants had previously been clinically diagnosed with a unilateral congenital transverse below-elbow limb deficiency. Unilateral congenital below elbow deficiency is also called transradial deficiency. Children with this condition typically have failure of formation at the proximal 1/3, distal 2/3 junction of the forearm. They do not have carpal, metacarpal, or phalangeal bones. All subjects met these inclusion and exclusion criteria, in addition to the inclusion criteria that their residual limb is long enough to support a prosthesis. All subject recruitment and experimental procedures were approved by the Shiners Hospitals for Children Western Institutional Review Board (WIRB). Written, informed consent was obtained from participants prior to participating in the study. For participants who were too young to provide informed consent, written informed consent was obtained from parents/guardians and participant assent was obtained.

**Table 1:** Subject information

Subject ID	Age	Sex	Limb classification	Affected Side	History of previously prescribed myoelectric prosthesis
A	10	M	UCBED	Left	No
B	6	F	UCBED	Right	No
C	17	M	UCBED	Right	No
D	18	M	UCBED	Left	No
E	20	M	UCBED	Left	No
F	8	M	UCBED	Left	No

**Table 2:** Participant limb dimensions

Subject ID	Affected limb		Unaffected limb	
	Length (cm)	Circumference (cm)	Length (cm)	Circumference (cm)
A	9	8.5	20	8.75
B	3	5	7	7.5
C	5	7	11	9
D	4.5	7	10	8.5
E	5.5	6	10	9.5
F	6.5	8.5	8	8.5

**Fig. 1:**
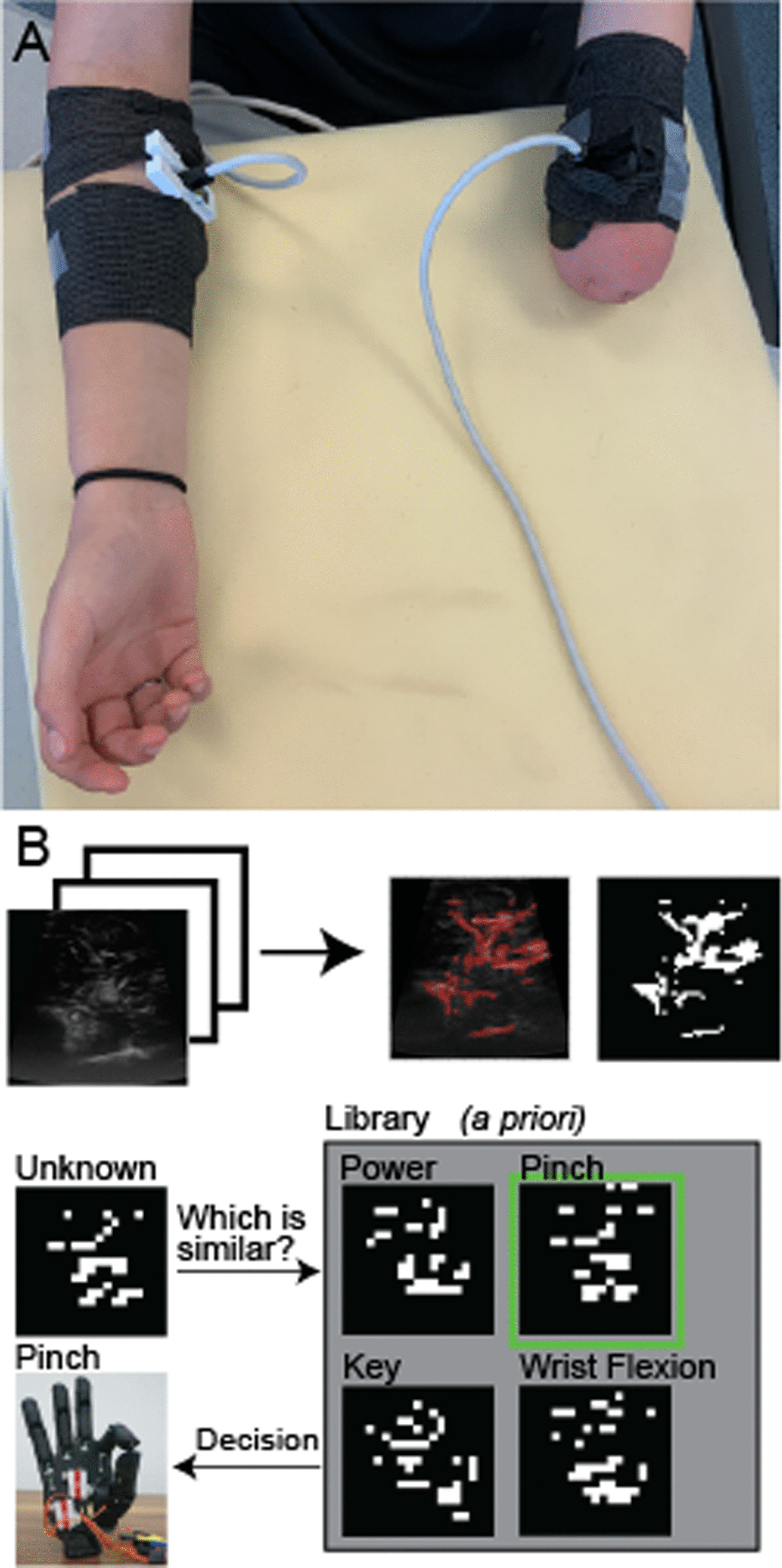
Representative placement of ultrasound transducer and visualization of machine learning algorithm. **A** Ultrasound transducers were attached to each limb of the participant with a 3D printed cuff and secured with self-adhesive Coban wrap. Ultrasound data was streamed directly from the Terason ultrasound system to a secondary computer for storage and offline analysis. **B** Spatiotemporal tissue deformations of each tested motion were filtered and scaled to create a feature set for the KNN algorithm. This supervised algorithm uses the known labels of the nearest known samples to classify an unknown sample

The testing procedure was similar for both arms (affected and unaffected). Participants were seated upright with their forearms resting on a table with their upper arm near parallel to the sagittal plane. A clinical ultrasound imaging system (Terason uSmart 3200 T, Terason, Tetratech Corporation) via a linear array transducer (16HL7 transducer, Terason, Tetratech Corporation) was applied to their arm and stabilized with a 3D printed support and Coban self-adherent tape (Fig. 1A). The ultrasound imaging depth was initially set to 4 cm without focusing. Imaging depth was adjusted based on the anatomy of each participant. The transducer was oriented over the ventral aspect of the forearm or residuum below the elbow. The transducer was adjusted to qualitatively maximize tissue deformation observed in the field of view while participants opened and closed their hand and attempted to open and close their missing hand. Ultrasound image data was transferred to a secondary PC (Intel i7-10750H, 32 GB RAM, 6GM VRAM NVIDIA GeForce RTX 2060) in real-time via a commercial video capture card (DVI2USB 3.0, Epiphan Systems, Incorporated) at 30 frames per second. The captured screen was then cropped to include only the relevant ultrasound image before being processed in MATLAB (MathWorks, Incorporated) using custom algorithms as described below (see Additional file 2: Movie S1 for exemplar trials showing synced real world and ultrasound videos). The de-identified aggregate data sets will be made available upon reasonable request to the corresponding author.

### Experimental protocol

We applied ultrasound imaging to predict hand and wrist motion intent in children with UCBED by measuring distinct spatiotemporal deformations of the forearm muscles associated with attempts to achieve specific hand grasps or wrist positions. The resulting final deformed configuration of the muscles as captured in the ultrasound imaging data (termed the end muscle state) can be identified by machine learning algorithms and used to predict hand and wrist motion intent [28, 29].

Participants were instructed to perform hand and wrist motions (power grasp, key grasp, point, pinch, wrist flexion, wrist pronation) simultaneously with both limbs, while recording ultrasound data from each limb (Fig. 1A). Since only one ultrasound system was used during testing, separate testing blocks were conducted while recording from each limb. The affected limb was always imaged during the first block to prevent any potential bias by practicing the movement while recording from the unaffected limb. During each trial, movements were self-paced but were required to be performed within a four second window. Between 5–10 trials were obtained for each motion. Participants were instructed and shown a motion by the experimenter after which the participant would perform a set of 5 trials before moving on to the next motion. If participants did not show mental or physical fatigue after 5 trials of each motion were completed, a second set of 1–5 trials was completed. As the full protocol took over an hour, the experimenter continually monitored for signs of mental or physical fatigue and selected the number of trials in the second set based on each child’s behavior. Previous work has shown that ultrasound imaging data in adults with limb differences can successfully train a KNN algorithm with as few as 5 repetitions per motion [29]. Therefore, motions were collected in sets of 5 trials to ensure that sufficient data for analysis was collected on the first pass of each performed motion in case the experiment had to be ended early for participant fatigue or if the child no longer wished to continue the experiment. For each trial, participants were asked to relax both limbs prior to imaging. The first image frame of each trial was considered the initial muscle state. We calculated a dissimilarity measure between each image frame to the initial muscle state, using the Pearson correlation coefficient (see Similarity Analysis below). Participants were instructed to maintain their hand state until the trial ended, after which participants were instructed to relax. The end muscle state was defined as the average muscle state for the five frames prior to the end of the trial. Image frames were downsampled from the raw image size of 1024 × 1024 pixels, to a 128 × 128 image. Pixels which did not deviate from their initial value across all trials were filtered prior to classification. These pixels primarily consisted of areas of the ultrasound system screen that did not contain ultrasound imaging data. Thus, the feature vectors used for our analyses were approximately 1000 pixels. While the data remain high dimensional, similar methods for ultrasound data have shown success in adults for both offline classification and real time control of a virtual device [28, 29].

### Classification analysis

**Fig. 2:**
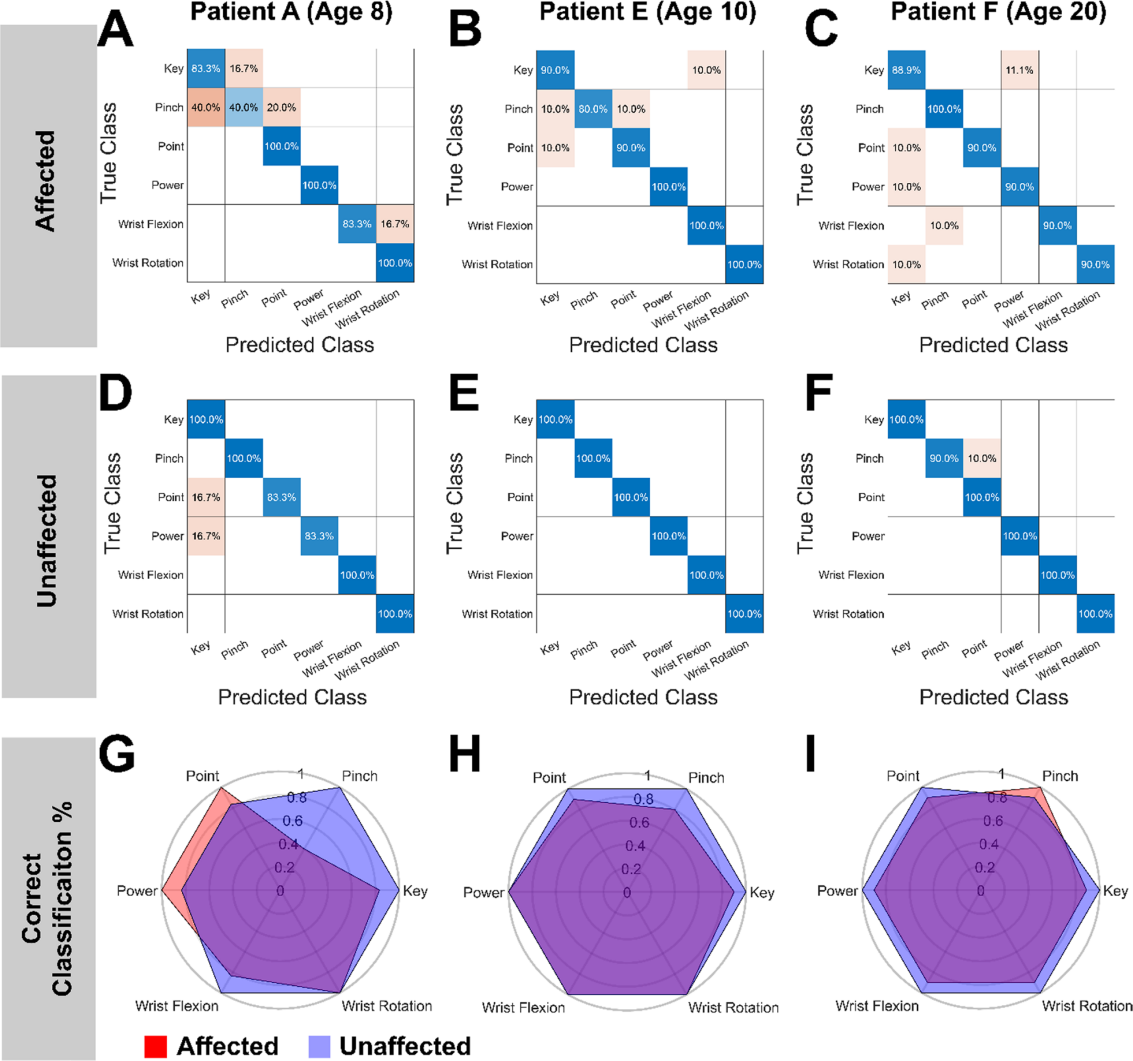
Confusion matrices and comparison of classification accuracy between limbs. Each column represents one of the three example participants (ages 8, 10, and 20, respectively). For most movements, performance between the affected limb (**A**, **B**, **C**) and unaffected limb (**D**, **E**, **F**) were comparable. Some participants had one poorly performing movement in their affected limb (e.g., the first example participant showed poor classification of pinch in their affected limb). The difference in classification accuracy between limbs is shown in **G-I**. The blue polygon represents the classification accuracy of the unaffected limb, while the red polygon represents the classification accuracy of the affected limb

A K-nearest neighbor (KNN) algorithm was used to classify the end muscle states of the six movement patterns. The only the end muscle states of the downsampled ultrasound images (Fig. 1B) were used for classification (Fig. 2). The Pearson dissimilarity measure between respective end muscle states was used as the distance metric in nearest neighbor classification. We used leave-one-out cross validation to calculate the classification accuracy and assess the performance of our classifier. Therefore, the KNN algorithm had between 4–9 known examples of each movement pattern, depending on the number of trials performed for each participant, during classification. As some participants had only 5 trials for individual motions, to maintain a consistent metric of performance, we considered 80% (4/5 trials correctly classified, see Table 3) classification accuracy (CA) the minimum acceptable threshold for reliable performance. This is slightly lower than the 85% CA threshold commonly used when evaluating machine learning classification accuracy in pattern recognition EMG system for adults [30].

**Table 3:** Subject trial information

Subject ID	Affected limb					
	Power	Point	Pinch	Key	Wrist Flexion	Wrist Rotation
A	5	5	5	6	6	6
B	7	7	6	7	7	7
C	9	9	9	9	8	9
D	10	10	10	10	10	10
E	10	10	10	10	10	10
F	10	10	10	10	10	10

Subject ID	Unaffected limb					
	Power	Point	Pinch	Key	Wrist Flexion	Wrist Rotation
A	6	6	6	6	6	6
B	7	7	7	7	7	7
C	6	9	9	9	9	9
D	10	10	10	10	10	10
E	10	10	10	10	10	10
F	10	10	10	10	10	10

### Similarity analysis

While classification accuracy is an excellent indicator of the extent the muscle end states of each missing hand movement can be distinguished from one another, it does not tell us how distinct a specific movement is from the others (e.g., pinch grasp compared to all other movements), nor does it tell us whether a movement is more or less distinct from the others when comparing between affected and unaffected limbs. Thus, we then applied a similarity analysis to the muscle end-states to complement our classification analysis findings. Similarity analysis, here termed Representational Similarity Analysis (RSA), has been used previously to examine fMRI and EEG activity patterns between brain regions and between species, where the underlying topology is not equivalent [31]. RSA uses the pairwise similarities between datapoints for comparison, rather than the datapoints themselves; thus, for two sources we can ask whether they represent a common set of stimuli in similar manners. This flexibility is critical for comparison between the unaffected and affected limb of children with UCBED; it cannot and should not be assumed that the musculoskeletal structures of the affected limb are equivalent to those of the unaffected limb. We constructed dissimilarity matrices for each limb to examine the informational structure of the muscle end-states. From these dissimilarity matrices, we calculated the exemplar discriminability index (EDI) of each limb of every subject to quantitatively assess the extent to which the tested movements are represented by distinct muscle states.

**Fig. 3:**
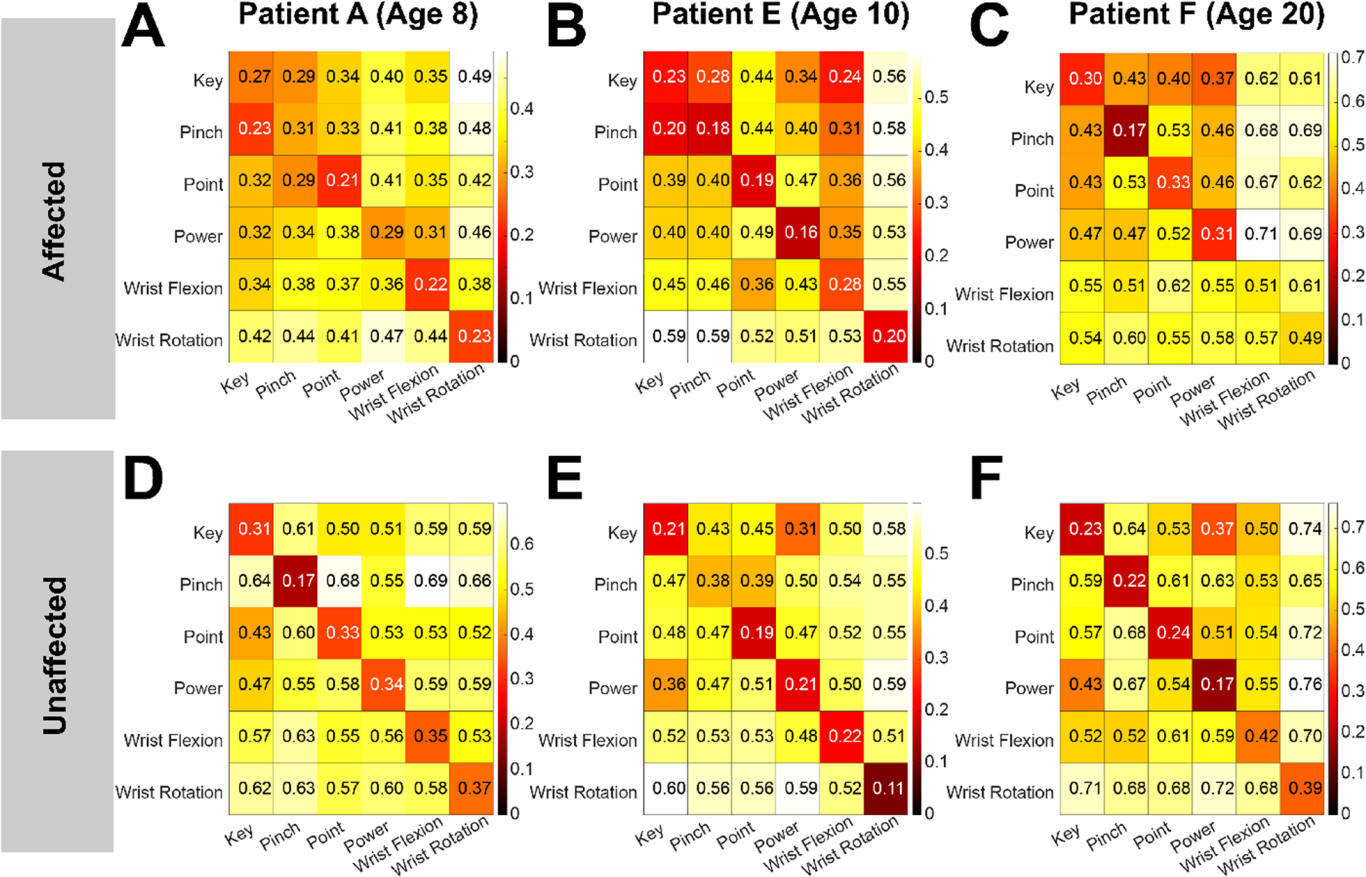
Split-data representational dissimilarity matrices. Results are for the same participants shown in Fig. 2 (separated by column), ages 8, 10, and 20, respectively. The top row (**A**, **B** and **C**) shows the results for the affected limb, and the bottom row (**D**, **E** and **F**) shows the results for the unaffected limb. In each panel the diagonal represents within-movement dissimilarity between the data-splits. The off-diagonals represent between-movement dissimilarities. As expected, most between-movement dissimilarity values are larger (lighter shading) than the within-movement dissimilarity values (darker shading). Our observation from Fig. 2, that the first example participant had poor classification of pinch in their affected limb is supported here; the within-pinch dissimilarity for their affected limb (**A**) is larger than the dissimilarity between pinch and key, as well as between pinch and point. We can also observe that poor dissimilarities between-movements are not restricted to the affected limb. The unaffected limb of the second example participant (**E**) shows fairly high within-pinch dissimilarity values, relative to the dissimilarities between pinch and all other movements.

It is critical to note that the pairwise dissimilarity values for any particular set of motions (e.g., power vs. point) cannot simply be compared between the affected and unaffected limbs, nor across participants, in isolation to assess whether the relation between the two motions is the same across limbs or across participants. It is the overall structure that is of interest here. It is not uncommon for dissimilarities to be transformed into a non-parametric measure (e.g., spearman’s rank) or converted first into percentile dissimilarity before visualization [32–34]. However, the experiment described here has only 6 categories (i.e., motions) and transforming to percentile dissimilarity would not be appropriate. As such, colors bars are shown on a participant by participant and limb by limb basis. All panels in Fig. 3 show their respective color bar to the right.

Finally, we used the temporal nature of our ultrasound imaging methodology to assess whether children were using a compensatory strategy to elicit multiple unique missing hand movement patterns. We compared the classification accuracy of our algorithm at trial start (initial muscle state) and trial end (end muscle state) to examine whether children might have biased their limb position between different hand and wrist motions. This would result in high classification accuracy even at the trial onset and obscure whether the children were volitionally performing distinct muscle patterns. Additionally, we applied multi-dimensional scaling on the full trials to examine the trajectories of each movement in ultrasound space. For each participant, within each limb, we took the pairwise distance between every frame of all trials and applied multi-dimensional scaling to visualize the muscle-state trajectories across all tested movements (i.e., power, point, pinch, key, wrist flexion, and wrist rotation). We qualitatively examined whether these trajectories shared a common path (e.g., the start of all power trials was the same position in the lower dimensional space as the end of point) which would be indicative of participants potentially using a compensatory strategy to elicit distinct muscle states.

### Statistical analysis

All statistical analyses were performed using MATLAB and Rstudio. All trials were manually checked by referencing the video recording of the unaffected limb of participants. Trials were removed if the participant did not perform the correct movement with their unaffected limb. On average, one trial was removed per movement per limb. Condition label randomization tests for each participant compared EDI measures across limbs with an alpha level of 0.05. The Pearson dissimilarity measure was used to calculate dissimilarity matrices of muscle end states.

## Results

### KNN machine learning can predict intended missing hand position from sonomyography data in children with UCBED

Figure 2A–C shows three representative subjects (ages 8, 10, and 20, respectively). In all figures where representative data is presented, complete data sets for all N = 6 participants with UCBED are provided in the supplementary materials. The results in Fig. 2A–C suggest that although they did not develop a hand, children with UCBED have robust motor control over the muscles of their residuum. For all six subjects, Leave One Out Cross-Validation (LOOCV) of our k-nearest neighbors (KNN) algorithm performed well above chance (chance accuracy = 16.7%) when simultaneously classifying all six missing hand movement patterns on data from participants’ affected limb. We then examined how many missing hand movements could be simultaneously classified with high accuracy, such that performance if implemented in a prosthesis would be reliable. As mentioned in the methods, we considered 80% classification accuracy (CA) the minimum acceptable threshold for reliable performance to maintain a stable metric across participants, due to our low and varying trial sample size (Table 3). All participants were able to achieve five simultaneously classifiable missing hand movement patterns with this high CA restriction. Two of our six participants were able to achieve 80% CA when simultaneously classifying all six missing hand movement patterns. Two of the subjects who were unable to achieve 80% CA in all six grasp patterns had Key grip as the worst performing movement pattern. This was not unexpected, as the primary differences between Power and Key grasps are thumb flexion and thumb palmar adduction vs. abduction. These thumb motions rely on intrinsic hand muscles and differences may be subtle when only imaging residual forearm muscles.

To put the results from participants’ affected limb into context, we also performed a LOOCV of our KNN algorithm on data from participants’ unaffected limb. Figure 2D–F shows KNN performance on the unaffected limb data from the three representative participants. Similar to the affected limb, our algorithm performed well above chance (chance accuracy = 16.7%) when simultaneously classifying all six missing hand movement patterns. KNN performance was well above 80% CA in all six participants.

Next, we compared performance between the affected and unaffected limb of each participant. To visualize this, we plotted the true positive rate of each limb as a polygon (Fig. 2G–I) with each vertex corresponding to a missing hand movement pattern and equally distributed angularly. For example, if a participant had 100% CA on all tested missing hand movements for their unaffected limb, the blue polygon would appear as a hexagon bounded at 1. Although performance of individual grasps is lower for some participants (e.g., participant A: pinch; participant B: key), overall performance is largely comparable between limbs in most participants.

### Missing hand movements are distinctly represented in the affected and unaffected limbs

We constructed split-data representational dissimilarity matrices (sdRDM) for each limb of every subject using the Pearson correlation distance as our similarity measure (Fig. 3A–F). Each dataset is equally divided, and an average muscle state is calculated as the average across trials in the respective half. We then calculate the pairwise distance between the estimated average muscle state of the two data splits. By converting these pairwise distances into matrix form, with the movements ordered the same direction along the vertical and horizontal axes, we obtain the sdRDM [32]. Thus, the diagonal entries provide the estimated dissimilarity between the same movements across the two halves and an estimate of the noise inherent to the data. The off diagonals reflect the dissimilarity between all other movement pairs. It is apparent that noise exists in the data, albeit less in the unaffected limb, as seen by the well above zero values of the diagonals, representing the within-movement dissimilarity. This suggests that there may be more motor variability in the muscle activations in the affected limb.

Quantitatively, we determined the extent to which the set of tested movements are represented by distinct muscle states, within each limb of each subject by calculating the exemplar discriminability index (EDI) from each sdDRM. This is a common summary statistic applied to RDMs in fMRI research, defined as the average between-exemplar dissimilarity minus the average within-exemplar dissimilarity [32, 35–42]. Within the context of single subject sdRDMs, we take the difference between the RDMs of the two data splits and sum along the diagonal to determine the within-exemplar dissimilarity, and across the off-diagonals to obtain the between-exemplar dissimilarity (see Additional file 1: Fig. S2). We performed an exhaustive enumeration label randomization test on the sdRDMs of each subject. Typically, this non-parametric test is performed over a specific number of iterations (e.g., 1000, 10,000); however, with only 6 movements in our experiment, there are only 720 possible permutations of the rows of our sdRMDs. Thus, we performed all possible label permutations. Under the null hypothesis of this test, we assume that the inter-movement and intra-movement average dissimilarities of the sdRDM are not significantly different and thus interchangeable (e.g., across the two data splits the average dissimilarity between power grasp in one half and power grasp in the other is the same as between power grasp in one half and point in the other). Therefore, under the null hypothesis the rows and columns of the sdRDM should be interchangeable. We calculated an EDI value for each permutation to obtain a null distribution of EDI values. The proportion of null EDI values that are greater than the actual EDI value can be considered the p-value for the test [32]. Due to the extreme heterogeneity of our participants, we performed this test on each subject and each limb separately to examine the representation of the movements. While this does allow for more isolated examinations in the case that any given subject has a unique result, it also does not allow for group level inference. We found that in all subjects, in each limb, the tested movements had distinct muscle state representations (Table 4). This supports our previous findings that our KNN algorithm was able to classify the tested movements in all subjects with well above chance accuracy. Additionally, we observe that while this is true, each subject had unique differences in the structure of their movement similarities. For example, Fig. 3B shows that for one subject, despite being able to classify key and pinch well above chance, their dissimilarity from each other is almost identical to their within-movement dissimilarity. That is, the muscle states of two split-halves for Pinch are as similar to each other as either is to the muscle state of Key from the other split-half. This suggests that despite being classifiable, key and pinch may not be as distinct, or well separated, as other movements for that subject. We can also observe certain commonalities across participants. As expected, wrist rotation was very dissimilar from every other tested movement in all participants.

**Table 4:** Exemplar discriminability indices and p-values for condition-label randomization test for all participants

Participant	Affected		Unaffected	
	EDI	p-value	EDI	p-value
A	0.121	< 0.01	0.263	< 0.01
B	0.140	< 0.01	0.293	< 0.01
C	0.081	< 0.01	0.242	< 0.01
D	0.069	< 0.01	0.413	< 0.01
E	0.228	< 0.01	0.282	< 0.01
F	0.196	< 0.01	0.328	< 0.01

### The similarity structures of the affected and unaffected limbs preserve many of the same limb movement muscle state relationships

**Fig. 4:**
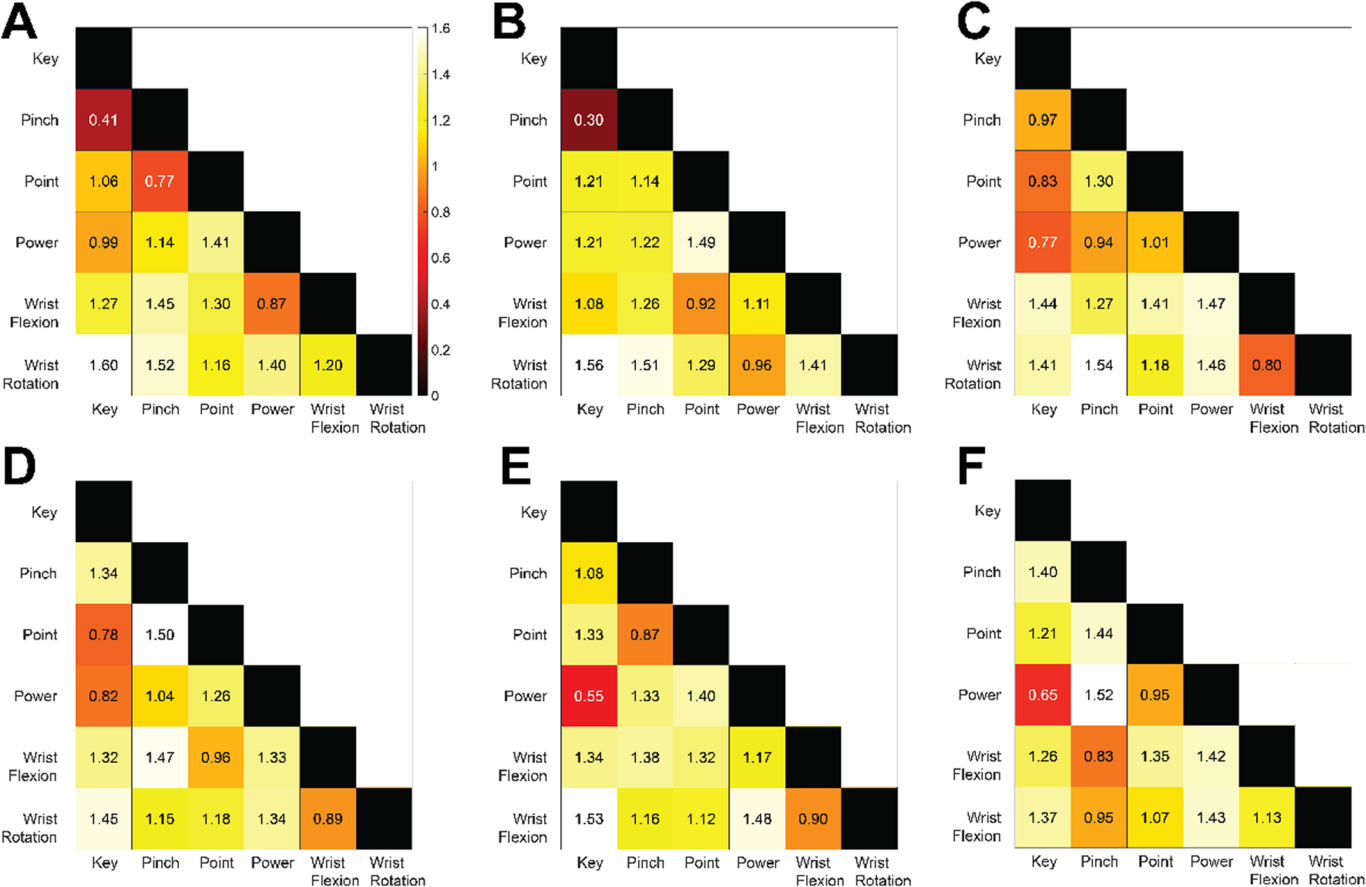
Representational dissimilarity matrices of the affected and unaffected limbs. Results are for the same participants as shown in Fig. 2 (separated by column), ages 8, 10, and 20, respectively. The top row (**A**, **B** and **C**) shows the results for the affected limb, and the bottom row (**D**, **E** and **F**) shows the results for the unaffected limb. In each panel the diagonal represents the similarity of the average muscle state for each limb movement to itself. The off-diagonals represent the pairwise similarity between each pair of average muscle states. As expected, wrist movements are very dissimilar from hand movements (lighter shading). Additionally, power and key showed low dissimilarity in the unaffected limb. Surprisingly, participant A showed low dissimilarity between point and key in their unaffected limb (darker shading). Post-hoc viewing of video recordings revealed unique thumb placement during point (palmar adduction) which resulted in a hand position more similar to key than other participants

To expand our findings that both limbs have distinguishable representations of hand, or missing hand, movements, we next compared the structure of how these movements are represented in each limb. Specifically, we examined whether the structure of how these limb movements are represented, within the given muscle architecture of each limb, is similar between limbs. That is, we quantified to what extent the set of relationships between pairs of muscle states (i.e., for each pair of limb movements) preserve similar patterns across limbs. Here, we constructed RDMs for each limb using the average muscle state across all trials of a given limb movement to calculate the similarity between pairs of limb movements. Thus, the RDM is symmetric around a diagonal of zeroes (Fig. 4). Similar to our previous analysis, we used an exhaustive enumeration condition label randomization test to compare the structure of the tested limb movements between limbs, within each individual participant. We calculated the Pearson correlation between the affected and unaffected limb RDMs as our measure of relatedness. Under the null hypothesis, we assume there is no relatedness of the structures of the tested movements between limbs. The rows or columns of one could be randomly permuted and the correlation between the two RDMs would not differ. Thus, to determine our null distribution, we calculated the correlation between every permutation of the labels of the unaffected limb and the unpermuted affected limb. The proportion of correlation values that are greater than the correlation between the true affected and unaffected RDMs can be considered the p-value for the test [32]. We found that in all subjects, there exists a statistically significant relationship of the structure of the limb movements between the affected and unaffected limb (Table 5). Critically, this suggests that despite the large differences in musculoskeletal architecture and experience using the muscles of each limb, both limbs represent motor intent of moving a hand in similar ways, within the topology of the given limb. Further, this suggests that the information related to motor intent present in the muscles of the affected limb may be much more robust than we have shown here. For example, we have shown that point and power (which differ by extension of the index finger in the unaffected limb) are highly dissimilar in the affected limb. Thus, it is possible that motor intent of moving the index finger itself may be derived from sonomyographic data of the affected limb.

**Table 5:** Correlation between the affected and unaffected limb RDMs and p-values for condition-label randomization test for all participants

Participant	Pearson correlation	p-value
A	0.77	< 0.01
B	0.63	< 0.01
C	0.68	< 0.01
D	0.68	< 0.01
E	0.78	< 0.01
F	0.85	< 0.01

### Purposeful modulation versus strategy

**Fig. 5:**
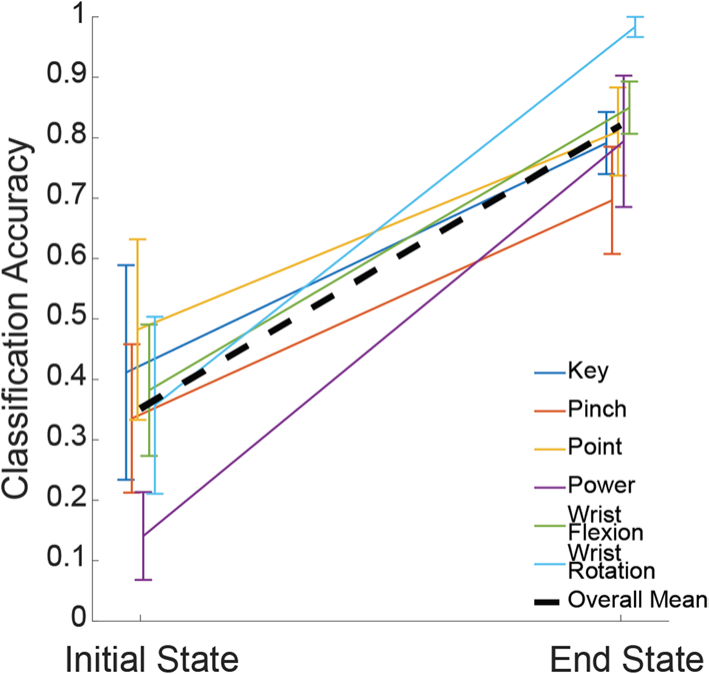
Classification accuracy at the start and end of trials. We observe a consistent trend of increasing classification accuracy between the start of every trial (Initial State) and the end of every trial (End State). The ultrasound images used in our KNN algorithm are again used here as our measure of the end muscle state for each hand and wrist motion. Shown classification accuracies for each hand and wrist motion are averaged across subject. Error bars denote the standard error of the mean for each hand and wrist motion. Overall Mean shows the classification accuracy of the initial and end muscle states averaged across both subject and motion

One major concern in the interpretation of the results was the potential for participants to utilize compensatory strategies to elicit unique muscle states for each movement pattern rather than attempting to move the missing hand into the prompted configurations. That is, while we expected participants to perform wholly distinct muscle contractions that resulted in unique muscle end states for each movement, it is possible to elicit unique muscle end states without requiring the preceding muscle contractions to be distinct. For example, a participant could have unintentionally altered their limb position between hand and wrist motions that could have biased the muscle states we used in our KNN algorithm. We observe a considerable increase in classification accuracy during the muscle activation across all tested hand and wrist motions in the affected limb (Fig. 5). This suggests that participants did not considerably bias the position of their affected limb between tested motions, and that the high classification accuracy seen in the end muscle states of the affected limb are due to volitional muscle movement.

**Fig. 6:**
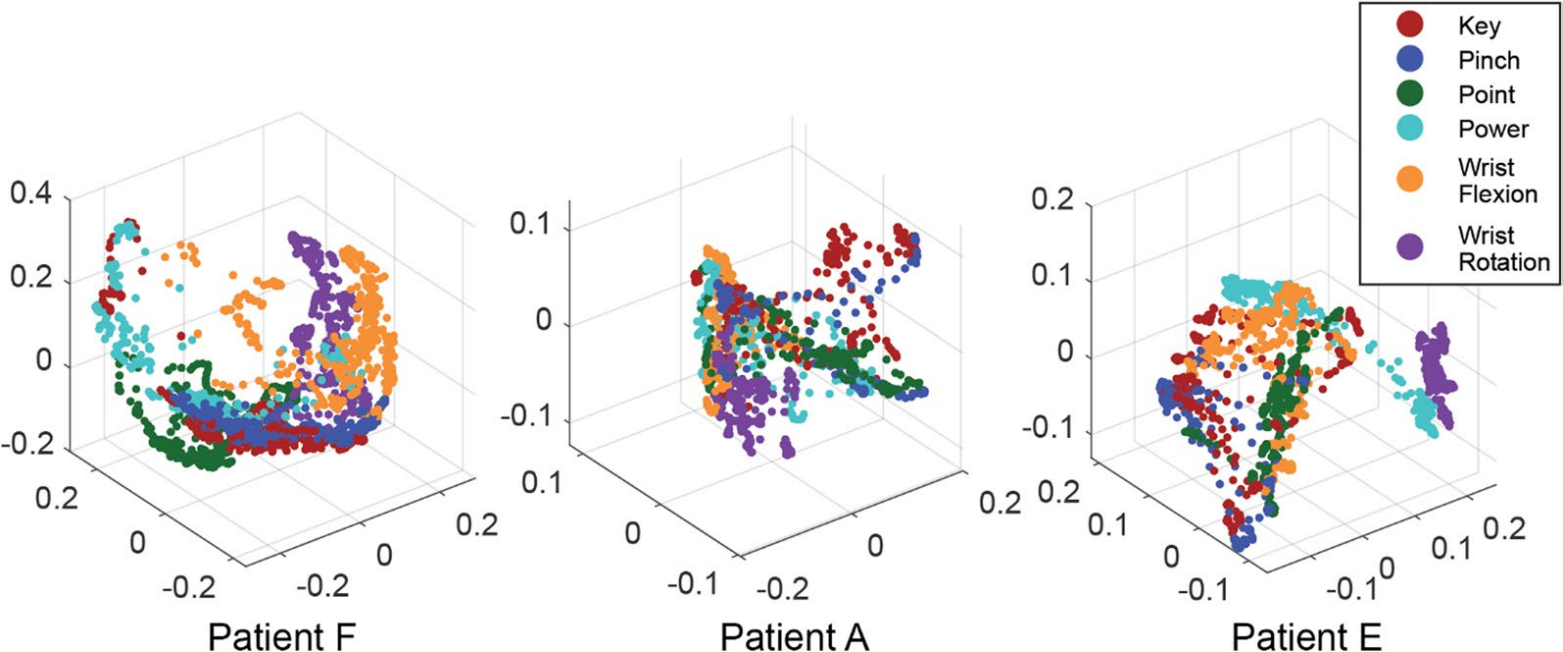
Multidimensional scaling of the pairwise distances of all tested movements. Results are for the same participants shown in Fig. 2 (separated by column), ages 8, 10, and 20, respectively. We used multi-dimensional scaling to visualize the trajectories of each missing limb movement in three dimensions. We observe that while movements often follow similar curvatures, they remain spatially separate. Furthermore, they do not align sequentially along a single curved path. The relative positions of each missing hand movement in this low dimensional space are highly subject dependent

Alternatively, a participant could hypothetically achieve multiple classifiable muscle states by skillfully using the same movement but activated to various intermediate positions. To examine whether such a compensatory strategy was used, we used multi-dimensional scaling to visualize the relative locations of each pattern. If such a compensatory strategy were to be utilized, a high degree of overlap in the muscle state trajectories would be expected. Figure 6 shows the pairwise distances of all tested movements, mapped onto 3 dimensions via multidimensional scaling, in the three representative participants shown in Fig. 2 (see Additional file 3: Movie S2 for rotating view of Fig. 6 for additional visualization). While power, point, pinch, and key grasps follow similar trajectories in the resulting low dimensional space, they remain spatially separate. This suggests that the previously hypothesized compensatory strategy was not used by participants, and they indeed performed distinct muscle contractions for each pattern.

## Discussion

Effective design of advanced prostheses requires a clear understanding of the motor control abilities of the user, and effective use requires a system that can reliably utilize information that spans the domain of the user’s motor control. This study demonstrates that children with UCBED have robust control of the muscles of their residuum and that this control can be accurately measured through ultrasound imaging (sonomyography) and machine learning. All participants were able to elicit simultaneously classifiable muscle states for at least five of the six tested movements in their affected limbs, and six of six in their unaffected limbs. We used a split-data RSA approach to quantitatively confirm that a statistically significant representation of the movements exists in both limbs of all subjects. Through these results, we show that there is an exciting opportunity to leverage children’s inherent motor abilities for prosthetic device control. This work lays the foundation to expand the functional repertoire of their devices, enhance the ability to maintain reliable performance, and ultimately impact the quality of life for this participant population.

### Implications for more intuitive prostheses

Understanding the extent to which children with UCBED can perform unique patterns of muscle activation is essential to inform the development of next generation upper limb prosthetic devices. The results presented here have shown that without any prior training, children with UCBED were able to consistently elicit distinct spatiotemporal muscle patterns for multiple hand and wrist motions both in the affected and unaffected limbs. Furthermore, we have shown that ultrasound imaging, coupled with machine learning, is able to reliably interpret motor intent from the activation of residual muscles, producing classification accuracies that could provide reliable control of multi-dexterous prosthetic devices. These results contrast previous work in sEMG systems for congenital limb difference populations, which have shown mixed success. For example, Kryger et al. which found classification accuracies of 52.1%$\pm$ 15.0% when using standard sEMG for classification of multiple hand configurations in adults with congenital upper limb deficiencies [24]. Additionally, Kaluf et al. which included four pediatric participants in their cohort while examining the abilities of persons with UCBED to use a pattern recognition sEMG system. The authors observed moderate success in calibrating their pattern recognition algorithm; 2/4 pediatric participants showed classification accuracy greater than 85% for 3 degrees of freedom [25]. While sEMG has served as a useful proxy for interpreting motor intent (i.e., muscle electrical activity), and has had great success in some populations, sonomyography provides an alternate measure of motor intent by observing the actual motor actions of the muscles (i.e., muscle displacements and deformations). We do not intend to suggest that ultrasound may supersede sEMG as a measurement modality for dexterous prosthetic control. However, our results support that ultrasound may be a promising modality that captures separate and relevant aspects of muscle motor control. As our ultrasound measurements showed a higher capacity for children to actuate their affected muscles than previously thought, we hypothesize that ultrasound-based control techniques may compliment established sEMG approaches. That is, a prosthesis control system that fuses both modalities may prove more reliable than either in isolation as together muscle activity and the driving motor intentions of individuals with UCBED can be more comprehensively characterized.

### Implications for the development of motor control

The size of our study sample limits the power to examine age-related changes in motor control of residual muscles in children with UCBED. However, our finding that children and adolescents aged 6–20 years have robust control of their residuum muscles is an encouraging indicator that examining age-related changes is possible. It has been well documented that motor control and multisensory integration during early childhood are not equivalent to that of adults [43–47]. Study of upper limb motor control in typically developing children has primarily focused on reaching tasks. For example, Wilson and Hyde observed that rapid online control improves, non-linearly, in children between ages 6–12 [45]. Similarly, others have shown that non-visually guided measures of motor control (e.g., force output and postural control) also change with age [48–50]. Therefore, it would be interesting to examine the extent such age-related changes in motor control also occur for residual muscles which have never actuated a hand.

It is important to emphasize that although some children in our sample have previous, although limited, experience with a myoelectric prosthetic device, they had no prior training in trying to explicitly mimic hand and wrist motions with their affected limb. Thus, the results presented here serve as a baseline assessment of the motor abilities of children with UCBED. It is likely that with training and practice there exists the potential for these children to improve the control of their residual muscles. Our findings that children with UCBED have robust peripheral motor control of their residual muscles and can elicit distinct muscle states when mimicking hand grasps may be surprising given recent fMRI work by Wesselink et al. This study found that adults with UCBED have no distinct cortical representation of the fingers of their affected limb [3]. The authors asked adults with UCBED to perform individual digit flexions or piano chord-like multi-digit flexions while recording from the primary motor and primary sensory cortices. It is possible that the cortical representation of residual muscles for persons with UCBED may not look similar to that of an unaffected hand or limb. Although we asked children to mimic the hand motions with their affected limb, it is also fair to argue that we are ascribing meaning to the muscle motions. It may be that the full set of possible muscle states a child can achieve with their affected limb is not analogous to the full set of muscle states for their unaffected limb. It is exciting to speculate on what the cortical representation exists for these unique residual muscle movements.

### Implications for other disease and injury conditions

The ability to study the control of the individual muscles, including deep muscles of the forearm, through sonomyography may be applied to additional clinical populations, and assessment of improvements in post-clinical intervention. For example, cerebral palsy is characterized by a combination of motor impairments, including excessive muscle co-activation [51–53]. However, it remains unclear whether the effect of excessive muscle co-activation is negative, or if it may aid in joint stability for participants who also exhibit muscle weakness. While surface EMG measurements have provided insight in characterizing muscle activity during movement in participants with cerebral palsy, sonomyography may prove a useful complementary tool for examining the spatiotemporal characteristics of motor control during grasping, particularly in the case of hemiplegic cerebral palsy. Our work here has demonstrated that sonomyography can serve as a useful tool in comparing the control of deep forearm muscles between limbs, even when the musculature does not necessarily behave typically. A combination of sonomyography and RSA could be used in comparing limbs pre and post intervention and could provide quantitative support on the extent spatiotemporal muscle patterns in the affected limb shifted towards those seen in the unaffected limb.

## Conclusions

In this study, we show that children with UCBED have robust control over the muscles of their residuum. When a child attempts to mimic movements with their missing hand, their muscles move in consistent and unique patterns for each motion. These spatiotemporal patterns carry information about their motor intent and can be classified by a machine learning algorithm. Critically, these motions did not need to be learned; participants were able to perform distinct patterns without any prior training or feedback on their performance. Furthermore, participants were able to perform distinct patterns regardless of their age or prior prosthesis history. Combined, these results are highly encouraging for the future of pediatric prosthetics. As more advanced prostheses become available, multimodal measurement technologies (including future ultrasound technologies) may provide clinically feasible control options that leverage the full capabilities of children’s affected muscles for more functional dexterous prostheses that provide significant improvement to participants’ quality of life.

### Supplementary Information

**Additional file 1: Figure S1.** Confusion matrices for all six UCBED participants. **Figure S2.** Example of how the EDI value is calculated.

**Additional file 2: Movie S1.** Spatiotemporal muscle deformation patterns and synced video of limb from a representative participant.

**Additional file 3: Movie S2.** Rotating perspective of multi-dimensional scaling of spatiotemporal muscle deformation patterns depicted in Fig. 6.

## Data Availability

De-identified aggregated data used in machine learning, similarity analysis, and MDS is available from the authors upon reasonable request.
