# Supplementary material for: Moving a missing hand: children born with below elbow deficiency can enact hand grasp patterns with their residual muscles
Source: J Neuroeng Rehabil. 2024 Jan 23;21:13. doi: 10.1186/s12984-024-01306-z (PMC10804465; doi:10.1186/s12984-024-01306-z)
Supplement: Supplementary file 1 — Additional file 1: Figure S1. Confusion matrices for all six UCBED participants. Figure S2. Example of how the EDI value is calculated. [file 12984_2024_1306_MOESM1_ESM.docx]

| \| 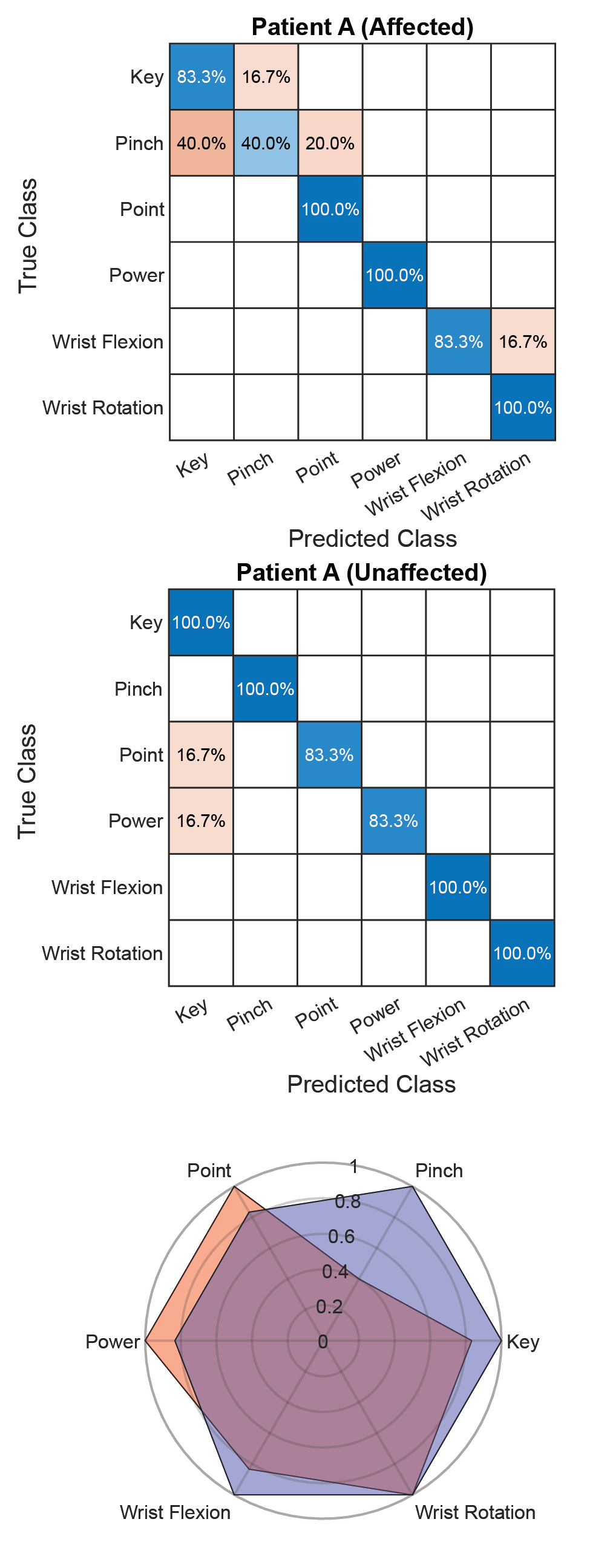 \| \| --- \| \| 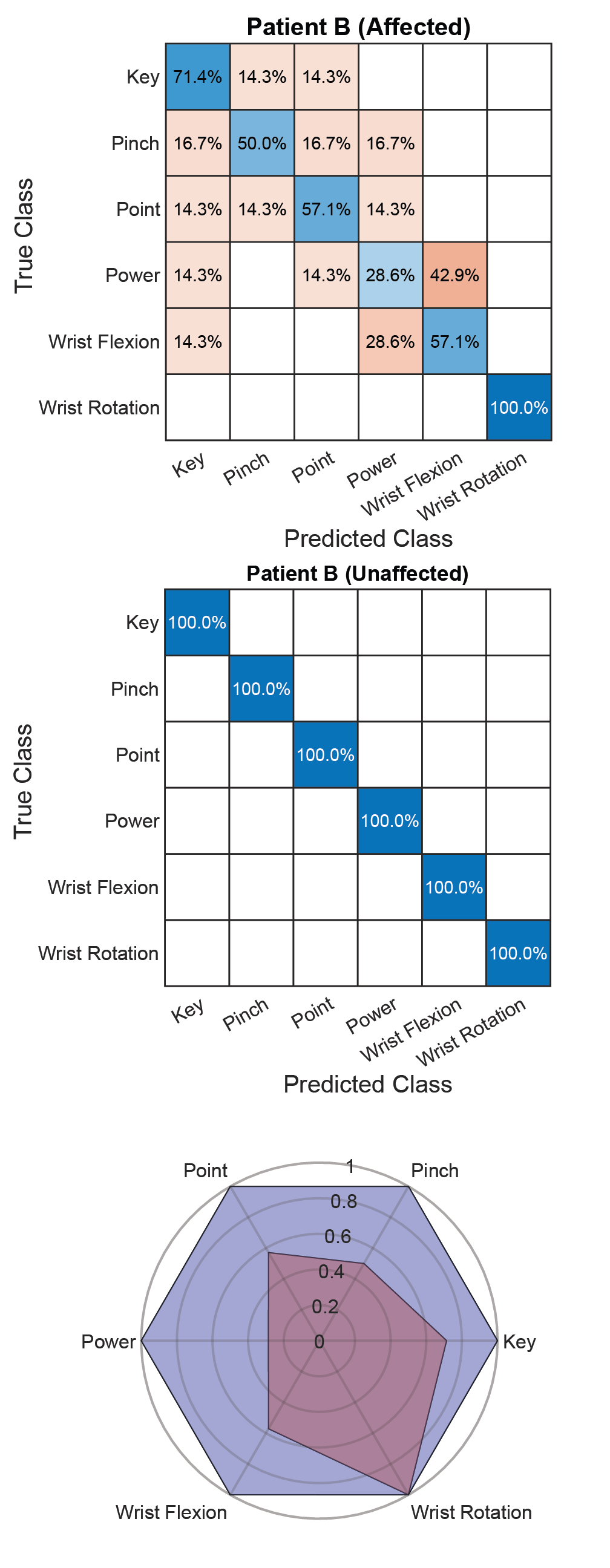 \| \| 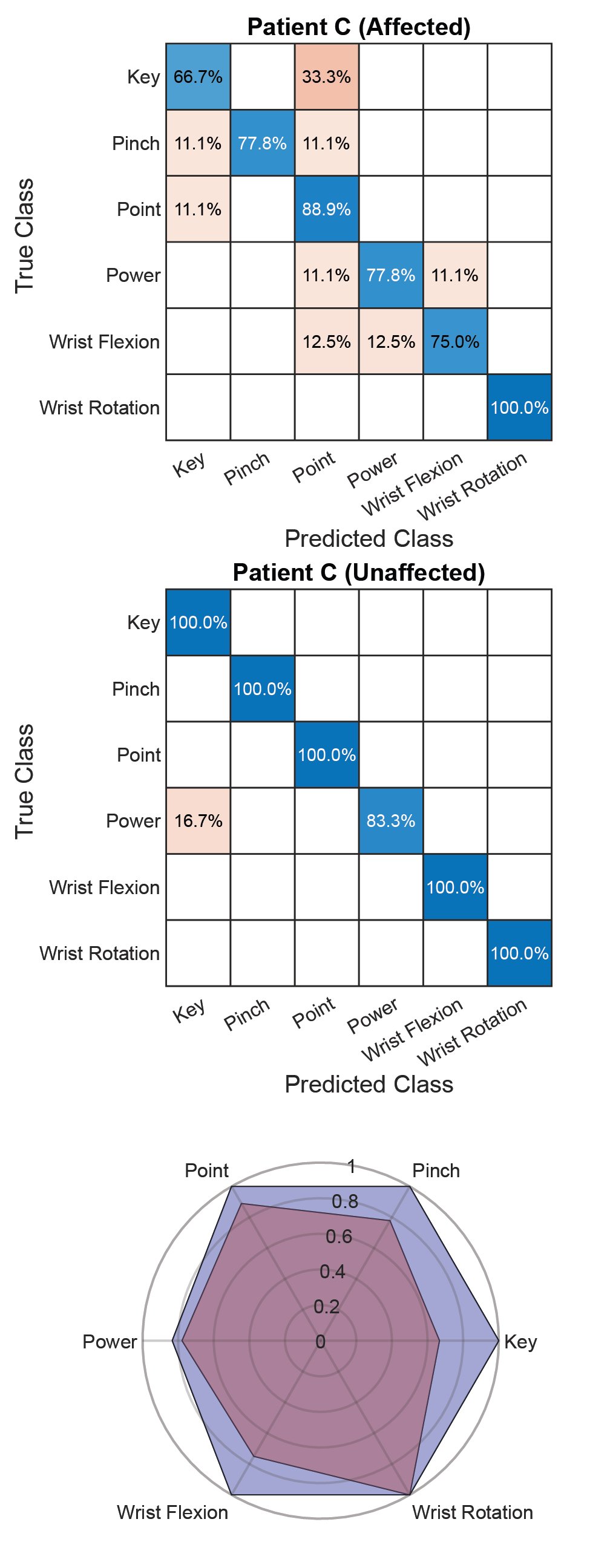 \| \| 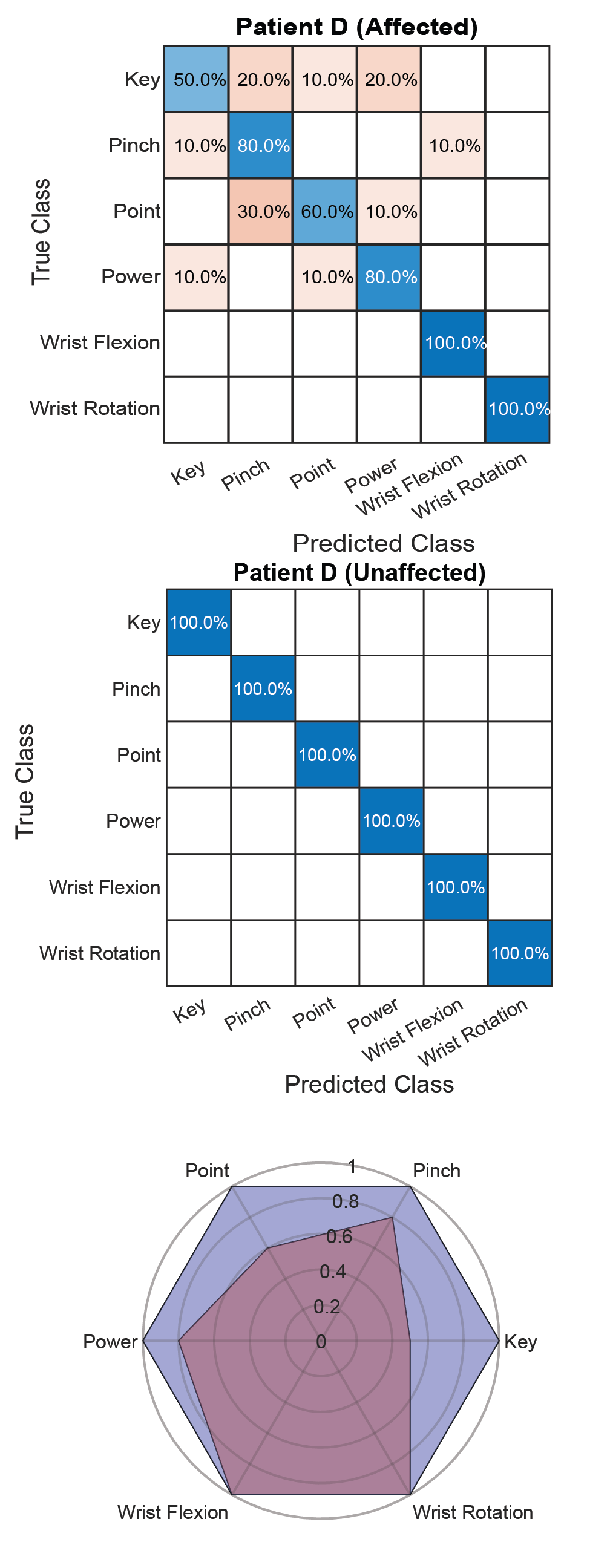 \| \| 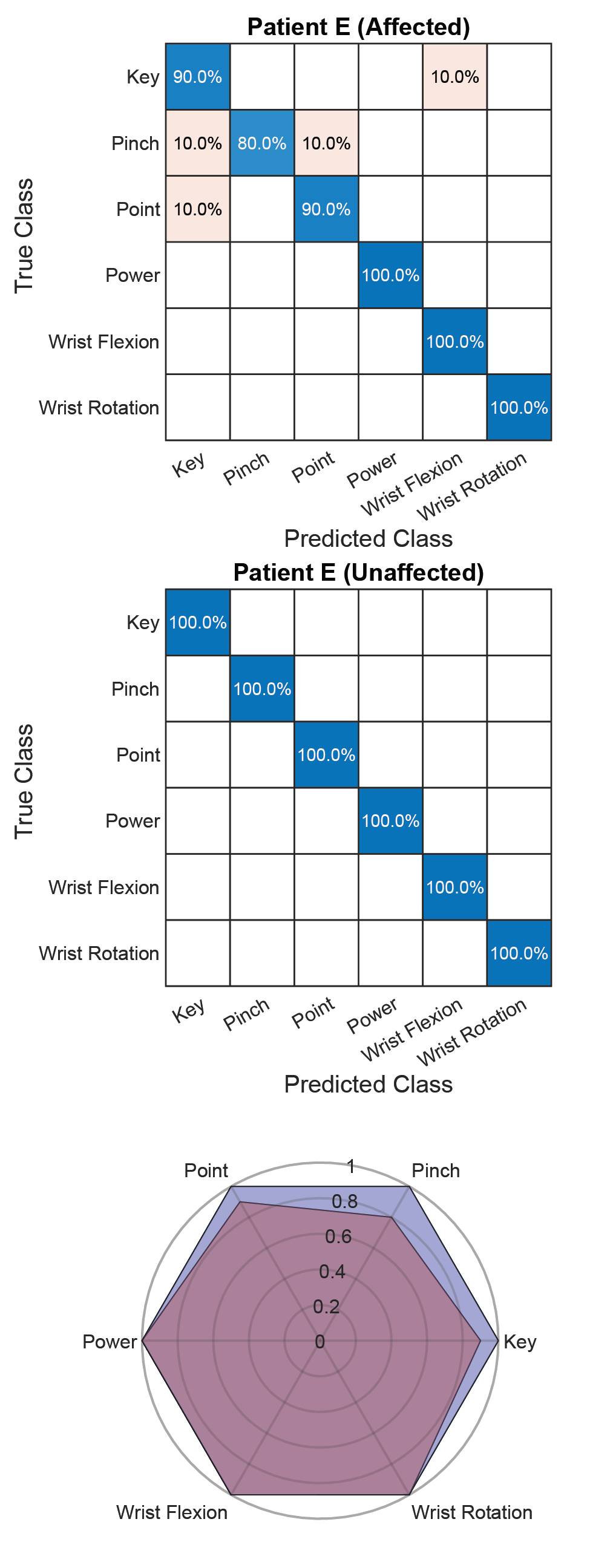 \| \| 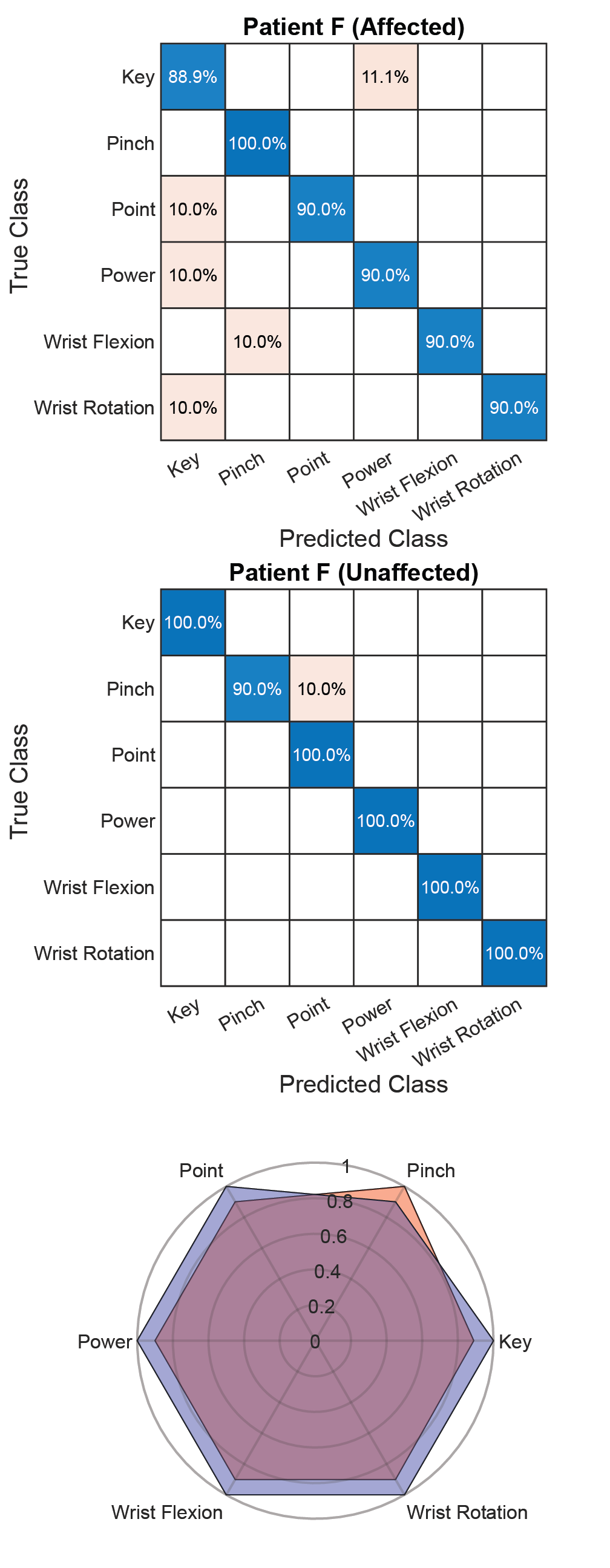 \| |  |
| --- | --- | --- | --- | --- | --- | --- | --- |
|  |  |
|  |  |

**Supplemental Figure 1. Confusion Matrices of Affected and Unaffected Limbs for all six patients.** The spatiotemporal muscle deformation patterns for all six movements had above chance accuracy in both limbs for all six patients. We observe that key, pinch, and point, tended to be misclassified more frequently across patients, but no consistent trend across all patients is seen. Power, wrist flexion, and wrist rotation are rarely misclassified across all patients. Three patients (patients A, C, and F) had misclassifications in their unaffected limb.

| **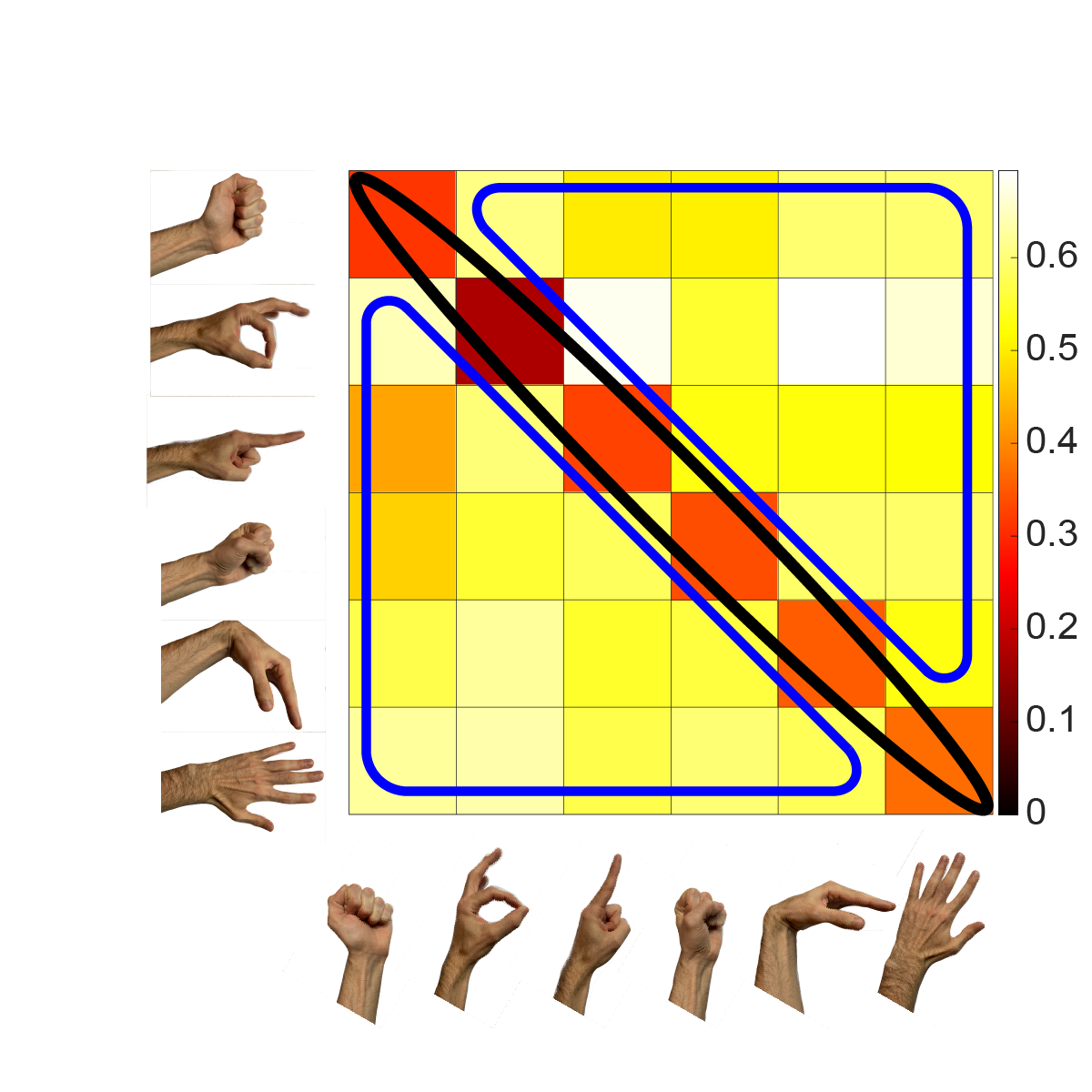** |
| --- |

**Supplemental Figure 2. How to calculate EDI.** The EDI value is calculated as the ratio between the average within-movement dissimilarity (black ellipse) and the between-movement dissimilarity (blue triangles). If the spatiotemporal muscle deformation patterns contain meaningful information about the missing hand movement intent, then we expect the within-movement dissimilarity to be much lower than the between-movement dissimilarity. Alternatively, if they do not contain meaningful information, there should be no difference and the EDI should be approximately 1.
